# Assessment of phytotoxicity of leachates from landfilled waste and dust from foundry

**DOI:** 10.1007/s10646-020-02197-1

**Published:** 2020-04-14

**Authors:** Marta Bożym

**Affiliations:** Opole University of Technology, Prószkowska 76 Street, Opole, 45–758 Poland

**Keywords:** Foundry waste, Dust, Heavy metals, Phytotoxicity, Germination index (GI), *Lepidium sativum*

## Abstract

The study assesses the contamination, classification and phytotoxicity of foundry waste. The presented results are a part of the research on the agrotechnical use of foundry waste. Landfilled foundry waste (LFW) and dust samples were taken from one of the Polish foundries. An analysis of the waste and its leachate composition was conducted. Phytotoxicity tests were carried out using *Lepidium sativum*. The aim of the phytotoxicity study was to evaluate germination and root growth after 72 h and the accumulation of heavy metals after 7 days. LFW was least contaminated with heavy metals and metalloids compared to dust. The composition of the foundry dusts depended on the unit of the foundry, from which it was collected. It was found that electric arc furnace dust (EAFD) was the most polluted by heavy metals among the dust samples. According to the requirements of Polish regulations most of tested waste were classified as non–hazardous, and EAFD as hazardous waste due to high Pb concentration in leachate. Phytotoxicity tests have shown a low phytotoxicity of the leachate from most of the tested waste. The results of the accumulation test showed that an excess of metal and metalloids in leachate was not directly related to its accumulation in plants. A negative correlation between EC, Cu, Co, Fe, Pb, Cr, K, Na, sulfate, fluoride, ammonia, phenol and formaldehyde concentration in leachate and GI was found. It was stated that the Fe, Mn, As and Se in plants was significantly correlated with concentrations in leachate.

## Introduction

One of the main directions of minimizing the negative impact of foundries on the environment is decreasing of waste generation and its reuse. Foundry waste mainly includes spent foundry sands (SFS). Moreover foundries also generate other waste such as dust and sludge from dust collectors, smelter slags, residue of refractory materials and spent grinding materials. About 600,000 tonnes of foundry waste are produced annually in Poland, including about 500,000 tonnes are waste from the iron foundry. Foundry waste, especially SFS, can be reused, e.g. in foundry production, in the construction and road industry. In addition, the use of SFS as a soil substitute is popular in the US and other countries (Dungan et al. 2006; Dayton et al. 2010). Foundry dusts can also be reused, e.g. in the production process of castings, in construction or to fill closed mines and excavations as inert material (Bożym and Dąbrowska 2012; Bożym 2018). The ability to reuse foundry dust depends on the composition and content of the contaminants. Dust is collected at various stages of casting production, e.g. while smelting metal in furnaces, cleaning casting and during SFS regeneration. Electric arc furnace dust (EAFD) is usually collected in a baghouse and amounts to ~2% of the steel produced (Strobos and Friend 2004). EAFD is one of the most hazardous industrial waste (Strobos and Friend 2004; Salihoglu and Pinarli 2008; Mymrin et al. 2016). This is related to the high content and potential leaching of heavy metals. In some cases it is profitable to recover metals from these dusts (Strobos and Friend 2004). EAFD can be reused in the production of castings and in metallurgy or for the production of Portland cement (Škvára et al. 2002; Mymrin et al. 2016). The EAFD can also be solidified with lime and concrete, in order to reduce the leachability of heavy metals, and landfilled (Andrés et al. 1995; Hamilton and Sammes 1999). The composition of dust from the regeneration unit depends mainly on the type of molding sand and binder. This dust is usually classified as inert and used in construction and road construction or due to the high content of residue of organic binder, it can be used as a fuel in cement plants (Bożym 2018).

The basic criteria for classifying waste for landfilling or for assessing applications for its use is the leachability of contaminants. The main contaminants in foundry waste are heavy metals (Bożym 2017, 2019) and organic compounds, formed as a result of the thermal decomposition of organic binders. Organic binders include phenolic, furan, phenol–formaldehyde, phenol–urethane, urea resins, furfuryl alcohol and other. For this reason, phenol, formaldehyde or cyanide may be present in foundry waste leachate (Lilja and Liukkonen 2008). In Poland waste classification is based on the quality of water extracts (leachate). Polish waste is classified as (1) inert, (2) non–hazardous and (3) hazardous (Journal of Law 2015). It turns out that physicochemical analyses are not enough to assess the toxicity of waste, because many contaminants can affect each other (García–Gómez et al. 2014b). Therefore, ecotoxicological methods are used, which indicate the impact on biota (García–Lorenzo et al. 2009). Widely used ecotoxicological methods include organisms from all levels of the food–chain, e.g. aquatic organisms for waste–water or leachate assessment (Quilici et al. 2004), earthworms for soil contamination assessment (Quilici et al. 2004; García–Gómez et al. 2014a; Curieses et al. 2016), snails for contaminated area assessment (Dallinger et al. 2004). One of the varieties of ecotoxicity tests is based on the use of plants, these are called phytotoxicity tests. These tests are mainly based on the assessment of apical endpoints such as germination, root growth and/or plant growth rate. In recent times, new protocols have been developed to determine the effects of toxins which are based on biochemical alterations, these are termed biomarkers. Among them, are biomarkers sensitive to heavy metals which have an impact on various biochemical processes in plants, e.g. enzyme and antioxidant production, oxidative stress protein mobilization and photosynthesis (Liu et al. 2009; Wang et al. 2010; Gupta et al. 2013; Alonso–Blázquez et al. 2015; Hu et al. 2015; Oztetik 2015; Rout et al. 2015; Seneviratne et al. 2019). In the phytotoxicity assessment of waste, leachate is usually tested, because nutrients and pollutants may be uptaken by the plants directly from aqueous solutions, in dissolved form. Contaminants in leachate can directly affect the germination of seeds and the development of seedlings, because plant growth depends directly on the medium. It is considered that seed germination bioassay could be less sensitive to toxic substances than root growth tests (Fuentes et al. 2004). Some authors suggest that toxic substances may not be absorbed by seeds, because the embryon (germ) of seeds is effectively isolated from the environment and draws nutrients (carbohydrates, macro– and microelements) from the seed (Mitelut and Popa 2011; Seneviratne et al. 2019). The roots are responsible for the absorption of water and nutrients, for this reason root lengths are affected by the concentration of the contaminants present in the water solution. The OECD guidelines (OECD 2006) provide many terrestrial plant species used to assess phytotoxicity. Mañas and De las Heras (2018) suggest that it is difficult to choose the appropriate plant species to analyse phytotoxicity. The authors propose that a general agreement should be reached between researchers in order to standardize this type of analysis. One of the most frequently used plants for phytotesting is garden cress (*Lepidium sativum*). The advantage of this species is the availability of seeds, rapid growth, sensitivity to environmental factors, including heavy metals, and low humidity requirements. *Lepidium sativum* is a recommended plant in many standards for biotoxicity test (Baumgarten and Spiegel 2004) and is used by many authors (Hoekstra et al. 2002; Fuentes et al. 2004, Barrientos et al. 2012; Baran et al. 2014; Visioli et al. 2014; Baran and Tarnawski 2015; Wierzbicka et al. 2015; Praveen et al. 2017; Khan et al. 2018; Mañas and De las Heras 2018; Seneviratne et al. 2019).

The aim of the study was (a) the analysis of the content of contaminants in the foundry waste and its leachates, (b) waste classification based on leachate quality, (c) a phytotoxicity assessment of leachates on the basis of germination and root length test, and (d) the assessment of the impact of leachate on plant growth and heavy metals, metalloids and nutrients accumulation.

## Material and methods

### Foundry

**Fig. 1:**
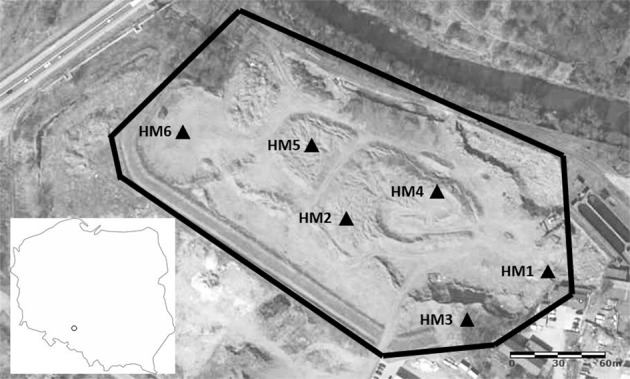
Location of the foundry and foundry waste landfill with sampling points (HM1–HM6). Source of maps: www.google.pl/maps

The foundry is located in south–west Poland (N 50°40′ 20.5853; E 18°12′ 44.7181″). The foundry produces parts for machine and industrial equipment from cast steel and cast iron. The foundry was the owner of two industrial waste landfills (heaps). According to data from 2003, 3.5 million tonnes of waste were landfilled on both heaps. At the beginning of the 2000s, a company which specializes in the production of road aggregates started waste recovery. Up to now, waste recovery at both heaps is being carried out. Metal, refractory materials, and parts of graphite electrodes are recovered. However, landfilled foundry waste (LFW) is mainly recovered and used for aggregate production. The main component of LFW are SFS. In addition, other foundry wastes are a component of LFW, e.g. slag, spent refractory material, metal inclusions, sludge and dust from dust collectors and sinters. Currently, the foundry owns one landfill. As a result of many years of waste recovery, this landfill has reduced its area from 8.8 to 4.7 ha. Figure 1 shows the location of the foundry and landfill. Additionally, sampling points at the landfill were presented. Foundry samples were taken from the waste landfill (LFW) and dust collectors from various foundry units.

### Sampling

LFW samples were collected in November 2017 from an industrial waste landfill, owned by the foundry. Samples were taken from six piles (*n* = 6), which were separated during material recovery, by a screening and sorting machine. Each cone–shaped pile was characterized by a height of about 3 m and a base diameter of about 3–4 m. Samples were taken from 4–5 places of each pile, from a depth of 25 cm, about 5 kg, creating a increment sample with a mass of about 25 kg. The increment sample was mixed and quartered to form a laboratory sample of ~5 kg. After transport to the laboratory, the material was dried at room temperature, sieved through a 4 mm (PN–EN 12457–2: 2006), ground in a mortar and again sieved to 1 mm.

The dust samples were collected in terms from 23.11.2017 to 12.01.2018 from the dust collectors of three foundry units: the forming unit, steelworks (induction furnaces) unit and shot blasting unit). Samples were given the appropriate numbers (P1–18). The characteristics of the dust sampling location, dust collector of forming unit: P1 and P18—shock grating dust (SGD), P2 and P16—regeneration of spent foundry sands dust (RD), P3 and P17—transport (of molding sands) dust (TD), steelworks unit: P4 and P14—electric arc furnace dust (EAFD); shot blasting unit: P5–P13 and P15—pneumatic blast cabinet dust (PBCD). Dust samples were not sieved. The foundry uses bag dust collectors and cassette dust collectors with pulse and jet filters. Dusts from the foundry are forwarded to external firms and used in metallurgy (EAFD and dust from pneumatic treatment of castings) or as filling in closed mines, as inert material (other dust).

### Phytotoxicity tests

#### Germination test

The germination tests were conducted based on the methodology of Zucconi’s test (Zucconi et al. 1981). The commercially available, certificated (PL–EKO–01) seed material of *Lepidium sativum* were used. The seeds were not pretreated. Twenty undamaged seeds of almost identical sizes were placed on a Whatman #1 filter paper in a 90 mm Petri dish, and 5 mL of waste leachate samples were added. Seeds were placed uniformly on the surface of a filter paper at the bottom of a Petri dish. As a control sample, Petri dishes were prepared as for leachates from foundry waste, only deionized water was added. The Petri dishes were covered and incubated at 20–25 °C in the dark. The number of germinated seeds was then counted and the elongation of the roots was measured after 72 h. The germination tests were conducted in triplicate, and the errors were recorded as standard deviations (SD). The germination index (GI) was calculated as RRG and RSG quotient up to 100% using Eq. (1) (Tiquia 2000):

1 $$GI{\mathrm{\% }} = \frac{{RSG \times RRG}}{{100}}$$

where, RSG – relative seed germination (%), RRG – relative root growth (%). RSG and RRG were calculated using Eqs. (2) and (3), respectively:

2 $$RSG{\mathrm{\% }} = \frac{{Gn}}{{Gc}} \times 100$$

where, Gn and Gc were the mean values of the number of seeds germinated in waste leachate and deionized water, respectively,

3 $$RRG{\mathrm{\% }} = \frac{{Ln}}{{Lc}} \times 100$$

where, Ln and Lc were the mean root length in the waste leachate and deionized water, respectively.

The root length was measured from the tip of the root to the radicle, with an accuracy of 1 mm.

#### Accumulation test

As part of the phytotoxicity tests of the foundry waste, an experiment on the accumulation of heavy metals, metalloids and nutrients in plants was carried out. The Petri dishes were filled with cotton wool and moistured with waste leachate (10 ml). The cotton wool was previously washed with deionized water for 24 h and dried at 105 °C. Then 1 g ± 0.1 g of *Lepidium sativum* seeds were added. Seeds were placed uniformly on the surface of a cotton wool at the bottom of a Petri dish. The experiment was conducted for 7 days. Every day, 1 ml of leachate or deionized water (control) was added to each dish, respectively. The dishes were kept at room temperature in the light (16 h) and in the dark (8 h). Every day of the experiment the growth and development of plants was observed. After this time the plants were separated from the cotton wool. The plant material was dried at 105 °C and grinding in a mortar. The dry mass of plants was mineralized and analysed. The accumulation test was carried out in triplicate for each waste sample.

Due to the adsorption properties of cotton wool, an experiment to determine the degree of pollution adsorption by cotton wool for each leachate was carried out. This experiment analogously to the accumulation test without sowing seeds was carried out. A correction factor as “cotton controls”, for each leachate has been included to provide free contaminants available for seeds. The percentage reduction of metal, nutrient and metalloid concentration in the leachate before and after adsorption by cotton wool is given in Table 1.

**Table 1:** ‘Cotton controls’ presented as correction factor [%] of metals and metalloids adsorbed by cotton wool from leachate

Metal (loid)	Cotton controls					
	LFW	SGD	RD	TD	EAFD	PBCD
Cd	n.c.	101	99	97	98	97
Pb	n.c.	93	90	97	95	95
Cu	95	93	92	94	93	91
Zn	96	98	99	96	85	88
Cr	86	92	83	90	84	86
Ni	98	95	92	n.c.	96	91
Mo	98	93	93	95	98	95
Co	96	93	89	95	94	n.c.
Fe	94	94	96	97	89	96
Mn	89	97	98	91	91	96
As	98	95	103	94	92	94
Sb	n.c.	n.c.	n.c.	n.c.	n.c.	n.c.
Se	n.c.	101	n.c.	99	97	100
Ca	85	85	84	89	91	86
Mg	94	88	87	91	91	89
K	94	91	98	92	91	93
Na	104	109	105	104	104	104

value < LOQ

*n.c*. not calculated

### Analysis of leachates

A leachate from LFW and dust samples were prepared in the ratio 1:10 (24 h) in accordance with PN–EN 12457–2: 2006. In leachates from waste and dust as well as in deionized water the following were determined: pH by potentiometric method (Elmetron CPC–501), electrical conductivity (EC) by the conductometric method (Elmetron CPC–501), sulfate(VI) concentration by the turbidimetry with barium chloride and the spectrophotometric method concentration of: chlorides with mercury thiocyanate, fluoride with SPADNS, formaldehyde with phenylhydrazine, phenol with phenyl hydrazine, cyanides with barbituric acid and pyridine, using UV–1601pc spectrometer (Shimadzu) according to Polish standards. Each sample was analyzed in triplicate.

### Heavy metals, metalloids and nutrient analysis

LFW samples were mineralized with *aqua regia* (9 ml 36% HCl, Avantor, and 3 ml 65% HNO_3_, Merck) according to the methodology ‘Soil’ (Millestone): 200 °C 10 min., 200 °C 15 min. (1000 W), 30 min. cooling. Dust samples were mineralized with 4 ml of HNO_3_ (65%, Merck), 3 ml of H_2_SO_4_ (96%, Merck) and 3 ml of HF (70%, Avantor) according to the method of ‘Saw dust with oil’ (Millestone): 200 °C 10 min., 220 °C 20 min. (1000 W), 30 min. cooling. Plant samples of *Lepidium sativum* were mineralized with 7 ml HNO_3_ (65%, Merck) and 2 ml H_2_O_2_ (30%, Avantor) according to the methodology ‘Dried plant tissue’ (Millestone): 200 °C 10 min., 220 °C 10 min. (1000 W), 30 min. cooling. The leachate were acidified with HNO_3_ (65%, Merck). Heavy metals (Cd, Pb, Cu, Zn, Cr, Ni, Fe, Mn, Mo, Co), metalloids (As, Se, Sb) and nutrients (Ca, Mg, K, Na) in the leachates and digest solutions were analysed using a Thermo atomic absorption spectrophotometer (Solaar 6) with FAAS technique. Each sample was analyzed in triplicate.

### Quality control and correlation analysis

For quality control of total heavy metals, metalloids and nutrients content in the leachate, mineralizates and plant samples, certified reference materials (CRM) mineralized in accordance with the above methodology were analysed. Due to the similarity of LFW to soils, soil CRM was used in the study. Four CRM materials were analysed: 1) ‘Metals in soil’ SQC001 (Merck), 2) ‘Urban particulate matter’ SRM 1648a (Sigma Aldrich), 3) ‘Fine dust (PM10–LIKE)’ (IRMM) ERM®–CZ120 and 4) ‘Lichen (trace elements)’ BCR–482 (IRMM). Recoveries obtained for metals and metalloids ranged between 90 and 115%. Additionally, a testing material (ICP multielement standard solution XI, Merck) to quality control of metal analysis in aqueous solutions was analysed. An analysis of variance (ANOVA) was used to determine significant differences (*p* < 0.05) between the control and tested samples. The correlation between the content of metals and metalloids in the leachate and *Lepidium sativum* and between the GI and the concentrations of various chemical constituents in the leachates was assessed using correlation matrices test and Pearson’s correlation analysis with STATISTICA version 13.3 software.

## Results and discussion

### Characteristics of waste

Table 2 shows the total content of heavy metals, metalloids and nutrients in the tested waste. The LFW were characterized by a low content of heavy metals and metalloids compared to dusts. The content of heavy metals in LFW was at the level of naturally occurring concentrations in soils around the foundry (Bożym et al. 2009). In the tested dust samples, including heavy metals and metalloids, a high content of Zn and Cu was found. EAFD samples were also characterized by a high Pb content (~2500 mg/kg DM). Due to their high content of heavy metals, EAFDs are most often classified as hazardous waste (Strobos and Friend 2004; Salihoglu and Pinarli 2008; Mymrin et al. 2016). In percentage basis, iron was the dominant component of the tested waste. The highest iron percentages were determined in PBCD and EAFD. The composition of PBCD is primarily affected by grinding shots (steel shots), used to clean castings. The composition of EAFD depends on the electric arc furnace input (steel scrap), because during the melting process, certain elements volatilize, and after cooling, these elements form a fine dust (Andrés et al. 1995; Hamilton and Sammes 1999; Škvára et al. 2002; Salihoglu and Pinarli 2008). The tested EAFD was also characterized by a high content of Mn. Other dust samples did not contain significant amounts of Mn. Among various nutrients, a high content of calcium and magnesium and a low content of sodium and potassium in LFW samples was determined. The source of Ca and Mg in LFW may be refractory materials and waste from furnace lining. A higher K and Na content was found in dust samples—SGD, TD and EAFD. The content of metalloids in all samples was low.

**Table 2:** Total content of metal/metalloids in foundry waste (mean ± SD)

Metal (loid)	Samples					
	LFW	SGD	RD	TD	EAFD	PBCD
Cd [mg/kg DM]	<0.2	<0.2	1.4 ± 1.3	7.0 ± 1.3	32.6 ± 6.5	1.0 ± 1.3
Pb [mg/kg DM]	33 ± 13	102 ± 73	31 ± 10	70 ± 21	2429 ± 92	13 ± 6
Cu [mg/kg DM]	78 ± 56	1024 ± 789	608 ± 84	384 ± 144	1208 ± 85	415 ± 205
Zn [mg/kg DM]	98 ± 24	1600 ± 267	900 ± 31	635 ± 43	3035 ± 568	5694 ± 4295
Cr [mg/kg DM]	118 ± 48	436 ± 77	252 ± 129	223 ± 29	201 ± 50	253 ± 191
Ni [mg/kg DM]	63 ± 27	160 ± 127	90 ± 4	63 ± 5	304 ± 57	579 ± 419
Mo [mg/kg DM]	27 ± 10	125 ± 13	69 ± 41	43 ± 13	136 ± 82	57 ± 42
Co [mg/kg DM]	17 ± 7	8 ± 3	7 ± 2	20 ± 3	37 ± 15	46 ± 23
As [mg/kg DM]	0.24 ± 0.07	13.84 ± 11.47	2.00 ± 0.42	0.56 ± 0.33	6.07 ± 2.86	1.51 ± 1.84
Sb [mg/kg DM]	1.16 ± 0.32	8.09 ± 5.90	2.19 ± 0.90	4.09 ± 1.73	3.94 ± 0.10	2.06 ± 1.58
Se [mg/kg DM]	0.10 ± 0.00	0.16 ± 0.03	0.21 ± 0.08	0.27 ± 0.18	0.22 ± 0.12	0.26 ± 0.22
Fe [%]	14.1 ± 1.0	17.7 ± 1.9	6.9 ± 3.1	12.9 ± 0.2	38.9 ± 3.4	49.3 ± 28.0
Mn [%]	3.38 ± 0.57	0.12 ± 0.06	0.06 ± 0.00	0.07 ± 0.01	7.44 ± 3.01	0.35 ± 0.16
Ca [%]	6.54 ± 2.11	0.44 ± 0.23	0.38 ± 0.33	0.10 ± 0.02	2.05 ± 0.13	0.69 ± 0.51
Mg [%]	1.61 ± 1.00	1.24 ± 0.58	0.60 ± 0.04	0.74 ± 0.09	2.86 ± 2.02	3.48 ± 1.62
K [%]	0.17 ± 0.06	3.44 ± 0.35	1.73 ± 0.89	1.81 ± 0.87	3.15 ± 0.20	1.27 ± 0.60
Na [%]	0.05 ± 0.01	6.01 ± 3.24	1.44 ± 0.23	4.46 ± 2.40	5.59 ± 3.29	1.26 ± 0.96

### Characteristics of the leachate

Table 3 presents the results of the leachate analysis of the tested waste compared to the Polish waste classification standard. The pH of leachate from the tested waste had a wide range, especially dust leachate: from acidic (pH 5.2) to alkaline (pH 9.2). LFW leachate was slightly alkaline (mean pH = 7.7). The EAFD samples were characterized by a range of pH, 6.5 (P4) and 8.6 (P14). Electrical conductivity (EC) is a parameter that determines the salinity of leachate. High salinity has a negative impact on the environment, especially on seed germination and plant growth. It turns out that plant resistance to environmental salinity depends on the species. Wang and Song (2019) found that low salinity (0.5% NaCl in soil solution) can reduce oxidative stress due to heavy metals in halophytes by increasing the levels of antioxidant enzyme activity. While Hoekstra et al. (2002) found that the salinity effects of leachate are not significant for plant germination at EC < 2000 µS/cm. The leachates from dust samples were characterized by a higher degree of salinity than the leachates from LFW, but they did not exceed the phytotoxic value. LFW leachate was characterized by a low EC value and thus low salinity. According to Polish law, the salinity of leachate from waste can also be determined on the basis of the sum of the chloride and sulfate concentrations (Journal of Law 2015). The chloride and sulfate concentrations were low in LFW leachate, they were below the inert waste limits. Leachate from the tested dust were characterized by a higher concentration of sulfates than LFW. Dust samples with the highest sulfate concentration were also characterized by a high EC value. This may indicate that the EC value of the leachate from the tested waste was primarily affected by sulfates. In contrast to chlorides and sulfates, fluorides are toxic to biota even at low concentrations (Stevens et al. 2000; Palmieri et al. 2014). Fluoride concentration was analyzed in the leachate, as it may be present in foundry waste. Organic binders are the main source of fluorides in foundry waste leachate (http://life-foundrysand.com). The fluoride concentration was low in all tested leachate. Plant resistance to fluoride depends on the plant species. Stevens et al. (1998) report the toxic for plants is fluoride concentration at 50–260 µM (1–5 mg/L) for tomatoes to 2532–13157 µM (50–250 mg/L) for cabbage. Gupta et al. (2009) found a negative effect of aqueous fluoride extracts on rice seeds on germination >20 mg/L and root elongation >30 mg/L, respectively. Although ammonia is not limited in Polish waste leachate, in the current study NH_4_^+^ content was analyzed, as potential source of eluted pollution. The main sources of ammonia in foundry waste are organic binders (Deng 2004; Siddiquea et al. 2010). It is known that ammonia cause a phytotoxic effect (De la Torre et al. 2000; Tiquia 2000; Hoekstra et al. 2002; Ramírez et al. 2008). In water extract, ammonia may be present in two chemical forms: NH_4_^+^ and NH_3_. The concentration of both forms depends on the pH. NH_3_ has been shown to be more phytotoxic (Hoekstra et al. 2002). NH_3_ concentration is calculated from the equilibrium equation from NH_4_^+^ concentration and pH. According to Bennett and Adams (1970) phytotoxic concentration of ammonia in water extract is 13 mM (≈220 mg/L). Whereas, Dowling (1993) found differences in tolerance to ammonia between plant species, e.g. the tolerance level for chickpeas was 20.8 mM (≈354 mg/L); for maize, wheat, barley, sorghum, panicum and sunflower was 10.2 mM (≈173 mg/L) and for cotton, canola and canary was 5.4 mM (≈ 92 mg/L). Based on the phytotoxic ammonia concentrations mentioned above, it may be concluded that ammonia concentrations in the leachate from the tested waste should not have a phytotoxic effect on *Lepidium sativum*. It should be noted here that the values given in Table 3 were converted from units of concentration mg/L to mg/kg (conversion factor ~10/1). No data for the phytotoxicity of ammonia to cress (*Lepidium sativum*) was found in the literature. The heavy metal concentration in the leachate was low (except EAFD), compared to the total content in the waste. The high concentration of lead in the leachate from EAFD was associated with a high total content of this metal in these dusts, which was confirmed by Škvára et al. (2002) in the analysis of leachate from EAFD. The lowest metal concentrations in the leachate were determined for LFW, which has been confirmed by studies of other authors (Hamilton and Sammes 1999; Škvára et al. 2002; Dungan and Dees 2009; Dayton et al. 2010; Siddiquea et al. 2010; Mymrin et al. 2016). An increased Fe content in the leachate from some dust samples (SGD, RD, EAFD) was found. Although the concentration of this metal is not normalized in waste leachate, it may negatively affect plant growth due to the inhibition of nutrient uptake and competition with other micronutrients (Rout et al. 2015). The concentration of metalloids in most of the leachate was below or within the limit of quantification. It follows that As, Sb and Se in foundry waste were in water insoluble forms. Arsenic, from all metalloids, is the most toxic pollutant (Kabata–Pendias 2010). The toxicity of arsenic is closely dependant on its chemical form. As has a negative impact on the root and shoot length of plants (Seneviratne et al. 2019). In contrast, selenium is a microelement which stimulates germination and plant development (Barrientos et al. 2012; Praveen et al. 2017). Antimony is an element whose influence on biota is not well researched and understood (Kabata–Pendias 2010). Sb toxicity to germination and plant growth depends on the species (Liang et al. 2018). Among the nutrients in leachate, the highest concentration of Na was determined. The leachate from EAFD was characterized by the highest concentration of all nutrients. Cyanides, phenol and formaldehyde from the residue of organic binders may be present in leachate from foundry waste (Oliva–Teles et al. 2002; Dungan et al. 2006; Siddiquea et al. 2010). These compounds are toxic to biota, therefore its leaching from foundry waste should be monitored (Siddiquea et al. 2010). However, it was found that phenol and formaldehyde from SFS may stimulate microbial activity (Dungan et al. 2006). There is a lack of information in the currently available literature concerning the effects of formaldehyde in aqueous solutions on seed germination and plant growth. In contrast, the influence of cyanide and phenol on plant germination is widely described (Hoekstra et al. 2002; Yu and Gu 2007; Palmieri et al. 2014). Hoekstra et al. (2002) found that cyanides and amines are nitrogen phytotoxic compounds that decrease plant germination. It is known that vascular plants have an enzyme system that detoxifies cyanides by converting them into the asparagine amino acid. For this reason vascular plants are used for the phytoremediation of soils contaminated with cyanides (Yu and Gu 2007). Palmieri et al. (2014) found that cyanides (0.0031 mg/L) may be less phytotoxic than fluorides (0.3938 mg/L) in elutes from solid waste from aluminum production (SPL—Spent pot–liner). In the current research, the cyanide content of LFW leachate was low compared to leachate from dust. The highest CN^-^ content was determined in the leachate from EAFD. The phenol content in the leachate from LFW, EAFD and PBCD was slightly higher than the limit value for inert waste. In contrast, the phenol content in leachates from SGD, RD, TD was very high (11.2–26.7 mg/kg DM). The source of phenol in foundry wastes may be phenol–formaldehyde binders which are used in the foundry. The tested leachates were characterized by a high concentration of formaldehyde. The highest formaldehyde content was determined in the leachate from SGD, RD and TD. The main component of these dusts are residues from spent binder and SFS.

**Table 3:** The characteristic of leachate from the tested waste, and classification based on limit values for the acceptance of landfilled wastes in Poland and the obtained values for tested waste (mean ± SD)

Parameter [mg/kg DM]	Classification of waste^a^			Landfilled waste samples	Dust samples				
	(1)	(2)	(3)						
				LFW	SGD	RD	TD	EAFD	PBCD
As	0.5	2	25	0.03 (±0.00)	0.24 (±0.10)	0.05 (±0.01)	0.10 (±0.08)	0.06 (±0.02)	0.05 (±0.05)
Cd	0.04	1	5	<0.05	0.53 (±0.53)	0.05 (±0.01)	0.10 (±0.01)	0.17 (±0.06)	0.12 (±0.11)
Cr	0.5	10	70	0.3 (±0.1)	0.9 (±0.7)	0.7 (±0.5)	0.4 (±0.1)	0.7 (±0.2)	0.5 (±0.4)
Cu	2	50	100	0.6 (±0.1)	7.4 (±1.7)	5.7 (±1.5)	2.4 (±1.6)	2.9 (±0.6)	1.0 (±0.7)
Mo	0.5	10	30	1.5 (±0.1)	0.9 (±0.1)	0.8 (±0.1)	0.6 (±0.1)	0.6 (±0.1)	0.5 (±0.1)
Ni	0.4	10	40	0.2 (±0.1)	0.6 (±0.3)	0.4 (±0.2)	0.2 (±0.0)	0.6 (±0.1)	0.5 (±0.3)
Pb	0.5	10	50	<0.5	5.6 (±5.6)	3.1 (±2.7)	6.8 (±5.7)	14.2 (±1.7)	2.0 (±1.2)
Sb	0.06	0.7	5	<0.1	<0.1	<0.1	<0.1	<0.1	<0.1
Se	0.1	0.5	7	<0.01	0.07 (±0.06)	<0.01	0.07 (±0.01)	0.08 (±0.07)	0.02 (±0.03)
Zn	4	50	200	0.5 (±0.1)	1.3 (±0.2)	0.8 (±0.2)	0.6 (±0.1)	2.2 (±1.2)	1.2 (±0.8)
Cl^−^	800	1500	25.000	396 (±168)	242 (±85)	242 (±169)	146 (±85)	1397 (±234)	10 (±0)
F^−^	10	150	500	4.9 (±1.4)	7.6 (±1.1)	8.5 (±0.2)	8.2 (±0.3)	8.6 (±0.1)	4.1 (±1.6)
SO_4_^2−^	1000	20.000	50.000	349 (±164)	12434 (±2102)	7855 (±1052)	6266 (±1306)	7715 (±1921)	143 (±62)
Phenol	1	n.r.	n.r.	1.8 (±0.4)	26.7 (±3.9)	20.1 (±2.8)	11.2 (±0.5)	1.4 (±0.2)	1.3 (±0.3)
Co	n.r.	n.r.	n.r.	0.2 ± 0.0	0.5 (±0.3)	0.8 (±0.1)	0.5 (±0.1)	0.4 (±0.1)	<0.2
Fe	n.r.	n.r.	n.r.	0.5 ± 0.0	4.3 (±0.7)	3.2 (±0.7)	1.0 (±0.5)	2.3 (±1.8)	0.6 (±0.2)
Mn	n.r.	n.r.	n.r.	0.6 ± 0.1	3.0 (±2.7)	1.4 (±1.0)	0.5 (±0.0)	6.1 (±1.6)	0.8 (±0.2)
Mg	n.r.	n.r.	n.r.	5 ± 2	5 (±4)	4 (±3)	7 (±1)	3 (±1)	2 (±2)
Ca	n.r.	n.r.	n.r.	4 ± 2	6 (±3)	4 (±0)	3 (±0)	45 (±2)	7 (±3)
K	n.r.	n.r.	n.r.	70 ± 17	257 (±205)	226 (±174)	490 (±461)	720 (±29)	45 (±19)
Na	n.r.	n.r.	n.r.	358 ± 99	430 (±37)	515 (±296)	543 (±17)	3343 (±938)	193 (±192)
NH_4_^+^	n.r.	n.r.	n.r.	3 ± 3	147 (±91)	201 (±159)	64 (±13)	57 (±25)	1 (±1)
Formaldehyde	n.r.	n.r.	n.r.	35.8 ± 12.7	118.7 (±4.1)	83.5 (±5.1)	68.9 (±6.4)	41.1 (±2.4)	29.2 (±8.6)
CN^−^	n.r.	n.r.	n.r.	0.02 ± 0.00	0.06 (±0.04)	0.10 (±0.04)	0.09 (±0.08)	0.14 (±0.13)	0.05 (±0.04)
pH	n.r.	n.r.	n.r.	7.7 ± 0.2	5.1 (±0.1)	5.4 (±0.5)	6.0 (±0.2)	7.6 (±1.5)	8.2 (±0.5)
EC [uS/cm]	n.r.	n.r.	n.r.	106 ± 29	1820 (±113)	1243 (±156)	1090 (±139)	1768 (±223)	43 (±16)

*n.r.* not required

^a^(1) inert, (2) non-hazardous, (3) hazardous waste (Journal of Law 2015)

In summary, according to the requirements of Polish regulations (Table 3) the LFW samples are classified as non–hazardous waste. The EAFD samples, due to the high concentration of Pb in the leachate, are classified as hazardous waste, although the concentration of other contaminants was below the requirements for non–hazardous waste. Other dust samples have been classified as non–hazardous waste.

### Phytotoxicity tests

#### The germination test

Seed germination rates (RSG) for all waste samples ranged between 90 and 100%. Therefore, RSG was not a sensitive indicator of phytotoxicity, a fact which has been confirmed by other authors (Fuentes et al. 2004; Gyuricza et al. 2010, Mitelut and Popa 2011). Differences were found in root length (elongation) (RRG) which affected the final GI value. The roots are responsible for the absorption of water, nutrients and contaminants, which affects the development of the plant. However, most of the nutrients are taken by the germ from the seeds, so the impact of contaminants on plant development in the first stage of growth may not be apparent (Khan et al. 2018; Seneviratne et al. 2019). During the germination test, different leachate effects were observed on *Lepidium sativum* seeds. An increased root length was observed for all leachates from LFW compared to the control. In a few cases, the tegument of seeds exposed to leachates from dust turned black (SGD, RD) and also for one sample of EAFD (P4). Leachate from these dust samples were characterized by low pH and a high concentration of Fe, phenol and formaldehyde (Table 3). A decreased root length was also observed for these seeds. A weak root elongation effect, without any change in tegument color, was also observed for leachate from one sample of EAFD (P14). The leachate from sample P14 was characterized by a high concentration of Fe (4 mg/L) and a slightly alkaline pH (8.6). Therefore, a possible cause of the blackening of the seed tegument may be high iron concentration and the low pH of the leachate. A similar effect of tegument blackening of Lepidium sativum due to iron in the water extract was obtained in another experiment.

**Fig. 2:**
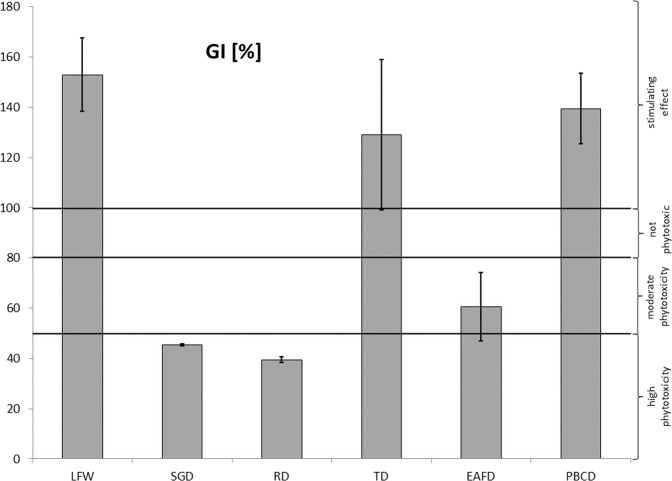
Germination index (GI) [%] of the foundry waste leachates. Error bars indicate standard deviations. The horizontal line represents limits on phytotoxicity/stimulating of root growth and seed germination expressed as GI

Figure 2 shows the GI values of all samples. GI values indicate the effect of the inhibition or stimulation of seed growth. The assessment was based on Zucconi et al. (1984) guidelines. An increase in root length and GI value compared to the control for LFW leachate was found, which indicates a stimulating effect on seed growth. Low concentrations of heavy metals and other contaminants were determined in leachate from LFW, compared to leachate from dust samples. Heavy metal concentrations were low and could stimulate germination and root growth (Seneviratne et al. 2019).These leachate were also characterized by neutral or slightly alkaline pH and a low EC value.

In the case of leachate from foundry dusts, significant differences in the length of *Lepidium sativum* roots were found. Lower root lengths and GI compared to the control were found for leachate from dust samples: SGD, RD and EAFD. The inhibition effect may depend on the high concentration of metals, and also on the interaction between the metals and other parameters (pH, EC, Cl, SO_4_, formaldehyde, phenol) of the leachate. Increased root growth compared to the control and a high GI value were found for leachate from other dust samples (TD, PBCD), which may indicate the stimulation of plant growth. The leachate from those samples were characterized by a low concentration of pollutants. It is known that many factors can affect root length (elongation) and GI such as unfavorable pH levels in combination with heavy metals, EC and anions, that may affect leachate phytotoxicity. Phytotoxicity is the result of a combination of several factors, which have inhibitory effects on plant growth. The metals in the leachates can have a dual effect, stimulation or inhibition, on the growth of seeds (Phoungthong et al. 2016). Heavy metals can inhibit plant seed germination and root elongation, when their concentrations exceed a certain value (Phoungthong et al. 2018). The pH value can negatively affect GI and root elongation only in the presence of other factors such as high heavy metal concentration. However, Phoungthong et al. (2016) found a negative influence of the pH of the leachate on GI only at a pH level which is strongly acidic, pH < 2 and a strongly alkaline pH, pH > 13. The authors stated that, the pH of leachate may be correlated with GI to a degree which is insignificant.

#### The growing and accumulation test

Plant appearance, cotyledon staining and plant height were evaluated. Poor coloring may indicate a lack of nutrients (Mg) in the leachate or the effect of heavy metals on chlorophyll. The central Mg atom of chlorophyll may be substituted by another divalent heavy metal in excess (Hg, Cu, Cd, Ni, Pb, Zn), which contribute to a decline in photosynthesis (Gupta et al. 2013; Seneviratne et al. 2019). In the accumulation test, as in the germination test, it was found that blackening of the tegument of *Lepidium sativum* occurred while it was grow on the leachate from SGD, RD and one sample of EAFD (No. P4). For these leachates, reduced *Lepidium sativum* sprout growth compared to the control and poor colouration of cotyledon were found. The reduced plant growth may be an effect of the high concentration of phenol, formaldehyde, ammonia and sulfates as well as the high EC value of those leachates. *Lepidium sativum* grew on the most of tested leachate, they were in good condition and characterized by well-colored (intensely green) cotyledons. The length of the tested plants (*Lepidium sativum*) growing on waste leachate, measured from the end of the roots to the cotyledon, ranged from 90 to 120 mm, with the exception of *Lepidium sativum* from SGD and RD leachate, which was only 7–13 mm in length. The average length of *Lepidium sativum* from the control was 120 mm.

**Fig. 3:**
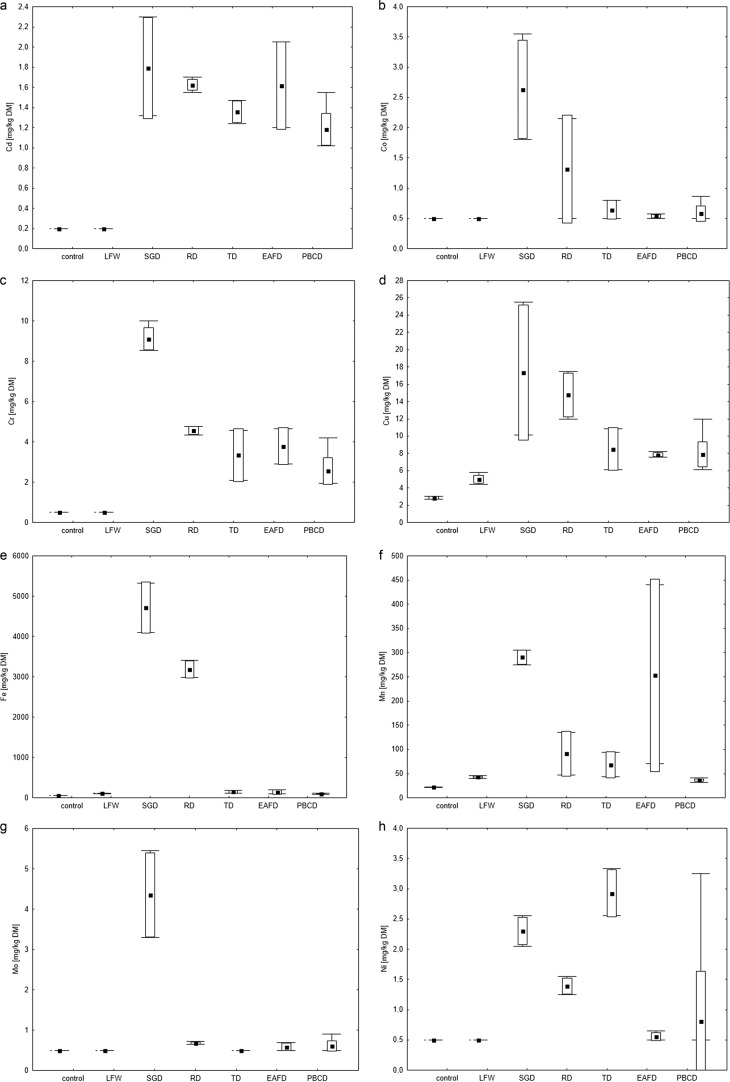

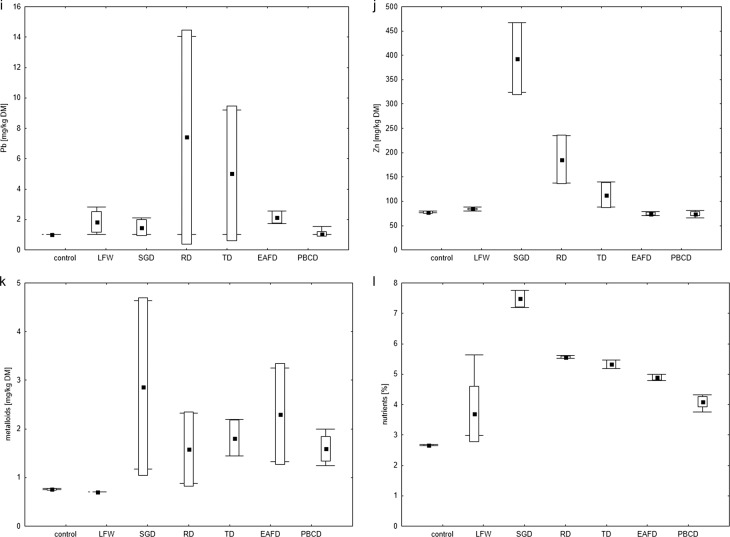
Box and whisker plots of heavy metals, metalloids and nutrients accumulated by *Lepidium sativum* after 7 days test, compared to control: **a** Cd, **b** Co, **c** Cr, **d** Cu, **e** Fe, **f** Mn, **g** Mo, **h** Ni, **i** Pb, **j** Zn, **k** the sum of metalloids, **l** the sum of nutrients. The edges of the boxes show the SD, the whiskers show min–max, respectively. The mean is marked with a square point

The total metal, metalloids and nutrients content in the *Lepidium sativum* is shown in Fig. 3. Among the metals analysed, the content of Fe and Mn was highest in the plants, which was due to the high concentration of these metals in the leachates. Among the other metals, the lowest content for Cd, Co, Mo and metalloids in *Lepidium sativum* was determined. The content of Cd, Ni, Mo, Cr, Co in *Lepidium sativum* from the control and LFW leachates was within or below LOQ. Plants (*Lepidium sativum*) growing on leachate with SGD and RD were characterized by the highest content of most metals, except Pb, compared to the control and other leachates. No high content of Pb in *Lepidium sativum* growing on leachate with EAFD was found, despite the high concentration of Pb in leachate from these dusts. In the case of RD and TD dusts, the Pb content in leachates differed significantly for the sample collected during the first and second terms. Similar differences which were found in the Pb content in *Lepidium sativum*, may indicate that the plants tested assimilated this metal from the leachate. Of the nutrients analysed, calcium and potassium from leachate were accumulated to the highest degree by most plants. Magnesium content in *Lepidium sativum* was slightly lower than Ca and K. Magnesium is the basic nutrient in the process of photosynthesis, as a component of chlorophyll, however, at the initial phase of growth (seedlings) photosynthesis is not intensive in plants. The accumulation of Na in the plants was low, despite its high concentration in the leachates.

In summary, in the discussion of accumulation by *Lepidium sativum*, it may be stated that the excess of a metal and metalloids in leachate was not directly related to its accumulation in plants. In the current research it the impact of other parameters affecting the accumulation of metals or metalloids by *Lepidium sativum* should be considered, not only the content of the leachate. It has been established that, heavy metals can be absorbed by plants passively (e.g. osmosis) or actively. Absorption is regulated by many factors, and plants have mechanisms that block the excessive absorption of metals from the substrate. Therefore, the content of metals in leachate is not necessarily correlated with its accumulation by plants.

#### Correlations analysis

The simple correlation between the content of heavy metals, metalloids and nutrients in plants and its concentration in the leachate and between GI and leachate variables were calculated (Table 4). A positive correlation means that both variables (element in the plant and element in the leachate) vary in the same way. A negative correlation implies that the relationship between the variables is the opposite, hence, when one increases the other decreases. In the correlation calculations, the limit value was used for the values below the limit of quantification. It was found that Fe (*r* = 0.871), Mn (*r* = 0.744), As (*r* = 0.893) and Se (*r* = 0.848) in plants was significantly correlated with concentrations in leachate. Lower correlation coefficients between for Cu, Cr and Co concentration in leachate and its content in *Lepidium sativum* were obtained.

**Table 4:** The Pearson correlation coefficients between the total content of metals, metalloids and nutrients in leachate from tested waste samples and *Lepidium sativum* tissue and between the constituents of leachates and the GI

	Content in *Lepidium sativum* tissue [mg/kg DM]																	GI
		Cd	Pb	Cu	Zn	Cr	Ni	Mo	Co	Fe	Mn	As	Se	Ca	Mg	K	Na	
Content in leachate [mg/kg DM]	Cd	0.142	−0.055	0.024	0.378	**0.475**^a^	0.253	**0.751**^b^	0.249	**0.535**^b^	**0.409**^a^	−0.067	−0.044	**0.534**^b^	−0.045	0.015	0.273	−0.349
	Pb	**0.517**^b^	0.324	0.354	0.207	**0.419**^a^	0.275	0.053	0.180	0.080	**0.481**^a^	**0.442**^a^	**0.583**^b^	0.230	0.171	**0.405**^a^	**0.497**^a^	−**0.453**^a^
	Cu	**0.525**^b^	**0.474**^a^	**0.645**^b^	**0.754**^b^	**0.827**^b^	**0.454**^a^	**0.749**^b^	**0.634**^b^	**0.910**^b^	**0.587**^b^	0.375	0.211	**0.825**^b^	−0.256	0.129	**0.749**^b^	−**0.776**^b^
	Zn	**0.483**^a^	−0.152	0.233	0.103	0.317	0.218	0.131	0.133	0.058	**0.490**^a^	0.304	**0.625**^b^	0.181	−0.067	−0.055	0.086	−0.347
	Cr	**0.439**^a^	−0.132	**0.731**^b^	**0.518**^b^	**0.547**^b^	0.145	0.218	**0.676**^b^	0.332	0.170	**0.598**^b^	**0.480**^a^	0.357	−0.103	0.137	**0.455**^a^	−**0.426**^a^
	Ni	0.276	0.061	0.001	0.042	0.240	−0.063	0.242	−0.036	0.195	0.320	−0.061	0.226	0.213	−0.139	−0.050	−0.011	−0.298
	Mo	−**0.798**^b^	0.040	−0.332	−0.029	−**0.436**^a^	−0.279	−0.045	−0.113	−0.036	−0.131	−0.360	−**0.612**^b^	−0.403	0.212	0.337	0.031	0.297
	Co	**0.506**^a^	**0.494**^a^	**0.751**^b^	**0.582**^b^	**0.516**^b^	**0.495**^a^	0.128	**0.592**^b^	**0.562**^b^	0.238	**0.515**^b^	0.365	**0.498**^a^	−0.244	0.136	**0.748**^b^	−**0.648**^b^
	Fe	**0.466**^a^	0.395	**0.620**^b^	**0.727**^b^	**0.750**^b^	0.370	**0.689**^b^	**0.621**^b^	**0.871**^b^	**0.489**^a^	0.330	0.154	**0.729**^b^	−0.234	0.261	**0.752**^b^	−**0.795**^b^
	Mn	**0.573**^b^	0.097	**0.435**^a^	0.339	**0.477**^a^	0.026	0.196	0.333	0.233	**0.744**^b^	**0.497**^a^	**0.715**^b^	0.271	0.084	0.363	**0.503**^a^	−**0.623**^b^
	As	**0.492**^a^	0.108	**0.732**^b^	**0.790**^b^	**0.752**^b^	**0.503**^a^	**0.617**^b^	**0.778**^b^	**0.568**^b^	**0.500**^a^	**0.893**^b^	**0.512**^a^	**0.652**^b^	−0.112	−0.066	**0.542**^b^	−**0.422**^a^
	Se	**0.616**^b^	0.038	**0.453**^a^	0.340	**0.511**^a^	0.297	0.180	0.367	0.110	**0.636**^b^	**0.537**^b^	**0.848**^b^	0.362	0.147	0.010	0.396	−0.352
	Ca	0.316	−0.063	−0.056	−0.158	0.134	−0.226	−0.057	−0.125	−0.134	**0.477**^a^	−0.048	**0.447**^a^	−0.099	0.206	**0.511**^a^	0.232	−0.388
	Mg	0.041	0.358	0.347	0.288	0.157	0.291	0.013	0.248	0.121	0.047	0.232	0.103	0.092	0.106	0.170	0.310	−0.036
	K	0.364	0.165	−0.032	0.033	0.225	0.205	0.136	−0.057	0.153	**0.553**^b^	−0.144	0.295	0.251	0.214	0.345	**0.435**^a^	−**0.546**^b^
	Na	0.223	−0.007	0.011	−0.047	0.124	−0.088	−0.036	−0.024	−0.040	0.343	−0.014	0.283	−0.060	0.220	**0.666**^b^	0.399	−**0.417**^a^
	pH																	**0.670**^b^
	EC																	−**0.847**^b^
	Cl^−^																	−0.344
	SO4																	−**0.828**^b^
	F^−^																	−**0.679**^b^
	CN^−^																	−0.343
	phenol																	−**0.723**^b^
	formald																	−**0.684**^b^
	NH4^+^																	−**0.701**^b^

The bold values represent statistically significant correlation

^a^Correlation is significant at the ≤0.05 level

^b^Correlation is significant at the ≤0.01 level

Negative correlation between Cu, Co, Fe, Mn and GI concentration was found. While, for Pb, Cr, K, Na and GI a low correlation coefficient was determined. Correlation coefficients between other metals, metalloids and nutrients and GI were not statistically significant. Negative correlations between metals and metalloids in leachate and GI was also found by other authors (García–Lorenzo et al. 2014; Phoungthong et al. 2016, 2018). A positive correlation was found between pH and GI for *Lepidium sativum*, suggesting that low pH value may be phytotoxic (García–Lorenzo et al. 2014; Phoungthong et al. 2016). In addition, the GI was negatively correlated with EC, which confirms that the increase salinity of leachate may negatively affects germination and root growth (García–Lorenzo et al. 2014; Phoungthong et al. 2016). Similarly to the EC, a negative correlation between sulfate, fluoride, ammonia, phenol and formaldehyde concentration in leachate and GI was found.

## Conclusions

LFW were characterized by a low total content of heavy metals and metalloids compared to dusts. EAFD was characterized by the highest content of Pb, Cd, Mo, Mn, Zn and Cu. Among the heavy metals, iron was the dominant component in all the tested waste samples. Among the metalloids, the highest content of Sb, followed by As was found in the tested waste. The Se content was the lowest of the metalloids. The content of nutrients (Ca, Mg, K, Na) in the tested waste varied within wide limits. The leachates from the tested waste were characterized by a wide pH range and salinity (EC). The highest concentration of pollutants in leachate from dust: SGD and RD were determined. These dust samples were collected from shock grating and installation of regeneration SFS dust collectors, and mainly consisted of residues of SFS and organic binders, which could be affected by high salinity, phenol and formaldehyde leachate from this waste. Leachate from LFW contained a lower concentration of pollutants than leachate from dust samples. According to the requirements of Polish regulations the tested waste samples were classified as: non–hazardous (LFW, SGD, RD, TD, PBCD) and hazardous waste (EAFD) due to high Pb concentration in leachate.

Higher GI values were found for LFW, TD, PBCD leachates compared to the control, which may suggest a stimulating effect of those leachate to *Lepidium sativum* root growth. Leachate from EAFD were characterized by a moderate phytotoxicity. In contrast, leachate from SGD and RD had phytotoxic effects on the germination and root growth of *Lepidium sativum*. The leachate from these dusts (SGD, RD) were characterized by low pH values, and high concentration of Cu, Fe, sulfates, phenol, formaldehyde and ammonia, compared to other leachates, which could have the effect of reducing GI value. For these leachates, a reduction in plant length and the poor coloration of cotyledon compared to the control along with a blackening of the tegument was also found, this may indicate the influence of one or more leachate components on these phenomena. In addition, the blackening of tegument phenomenon was also found for one sample of EAFD dust, which was characterized by low pH and an increased Fe content in the leachate. This effect was not observed for the second EAFD sample, which was alkaline (pH = 8.6). For this reason, it was stated that both of these factors, i.e. high Fe concentration and low pH, may affect the blackening of tegument. Current studies have concluded that the excess of a metal and metalloids in the leachate was not directly related to its accumulation in plants. One example was the low Pb content of *Lepidium sativum* growing on leachate from EAFD contaminated with this metal. Based on the correlation analysis, it was found that the plants may accumulate metals such as Fe, Mn, As and Se from leachate. For other components lower statistically significant correlation was calculated. In terms of the relationship between GI and parameters of the leachate it was found that EC, Cu, Co, Fe, Mn, Pb, Cr, K, Na, sulfate, fluoride, ammonia, phenol and formaldehyde concentration were negatively correlated and pH positively correlated. In summary, in order to assess the accumulation of elements by plants the impact of many parameters should be considered, and not only the content in the leachate. The plant may inhibit the absorption of pollutants from the substrate through the activation of absorption blocking mechanisms.

## Supplementary information

Supplementary Information
